# GraphChrom: A Novel Graph-Based Framework for Cancer Classification Using Chromosomal Rearrangement Endpoints

**DOI:** 10.3390/cancers14133060

**Published:** 2022-06-22

**Authors:** Golrokh Mirzaei

**Affiliations:** Department of Computer Science and Engineering, Ohio State University, Marion, OH 403302, USA; mirzaei.4@osu.edu

**Keywords:** chromosomal rearrangement, cancer classification, graph neural networks

## Abstract

**Simple Summary:**

Cancer is among the leading causes of death in the United States and worldwide. Early prediction of cancers is important for the improvement of treatment outcomes and survival rates, thus resulting in significant social and economic impacts. Recent developments have focused primarily on using gene expression and mutation data to predict or classify cancer types. Here, we show that chromosomal rearrangement endpoints alone can predict cancer types with more reliability and specificity.

**Abstract:**

Chromosomal rearrangements are generally a consequence of improperly repaired double-strand breaks in DNA. These genomic aberrations can be a driver of cancers. Here, we investigated the use of chromosomal rearrangements for classification of cancer tumors and the effect of inter- and intrachromosomal rearrangements in cancer classification. We used data from the Catalogue of Somatic Mutations in Cancer (COSMIC) for breast, pancreatic, and prostate cancers, for which the COSMIC dataset reports the highest number of chromosomal aberrations. We developed a framework known as GraphChrom for cancer classification. GraphChrom was developed using a graph neural network which models the complex structure of chromosomal aberrations (CA) and provides local connectivity between the aberrations. The proposed framework illustrates three important contributions to the field of cancers. Firstly, it successfully classifies cancer types and subtypes. Secondly, it evolved into a novel data extraction technique which can be used to extract more informative graphs (informative aberrations associated with a sample); and thirdly, it predicts that interCAs (rearrangements between two or more chromosomes) are more effective in cancer prediction than intraCAs (rearrangements within the same chromosome), although intraCAs are three times more likely to occur than intraCAs.

## 1. Introduction

Most cancers develop due to accumulation of mutations in somatic cells, although hereditary mutations do contribute to some cancers. Structural chromosomal rearrangements, also called chromosomal aberrations (CA), constitute translocations, deletions, inversions, duplications, and other forms of fusion of long chromosomal segments and have been detected in nearly every analyzed cancer genome. Aberrations could affect one single gene as well as entire chromosomal segments and generally result from improper repair of double-strand breaks in DNA. CAs alter genome architecture which can affect cell cycle regulators and cause cellular transformation and immortality. Additional complexity in terms of association between the arrangements may be developed when a significant portion of CAs occurs in one cell. Li et al. [1] described somatic structural variations in whole-genome sequencing and used spatial and temporal proximity to cluster structural variants.

Current genetic cancer theories propose that gene-level aberrations such as mutations are drivers of cellular immortalization and cancer. However, chromosomal instability, both structural and numerical, has been detected in virtually every cancer type [2]. Some proponents of the chromosomal theory of cancer have suggested that chromosomal instability may be the major driver [3]. Several studies revealed the significance of chromosomal rearrangements in cancer. Koschni et al. [4] found that comparative genomic hybridizations (CGH) have unique aberration patterns in oligodendrogliomas [4]. More specifically, the aberration pattern in subgroup C is different from that in subgroups A and B, which indicates a unique molecular carcinogenetic pathway of this subset of oligodendrogliomas. Heng [5] described the genome chaos and microcellular evolution and emphasized the higher importance of nonclonal chromosomal aberrations and genomics over genetics. We also identified cancer-specific chromosomal rearrangements [6]. Thus, it is becoming apparent that both mutations and chromosomal instability contribute to cellular transformation.

Cancer classification is a vital problem that helps clinical treatment and therapy. The main goal of cancer classification is to use somatic mutations to classify cancer into one of the cancer types or subtypes. Cancer prediction and classification have been investigated using different methods. The classifications based on morphological characteristics lack accurate diagnosis and may have a strong bias in diagnosis by experts. With the emergence of RNA sequencing, gene expression data, and whole-genome sequencing, more opportunities have arisen to examine the global landscape of mutations and discriminate between cancer types more accurately. Previous works in the area of cancer type/subtype classification used mostly gene expression data or plasma cell-free DNA methylation patterns. The gene-based cancer type classification approach described by Jiao et al. [7] can discriminate between 24 cancer types based on somatic passenger mutations.

Additionally, advances in machine learning and, more recently, deep-learning techniques have led to development of more accurate methods for diagnosis and treatment of cancer. Several machine learning-based approaches are proposed for cancer prediction and classification based on the support vector machine (SVM) [8,9,10,11], vanilla neural network [8,12,13], ensemble convolutional neural network [8,14], ensemble random forest and deep neural network [15,16], coherent voting network [17], and k-nearest neighbors (kNN) [18,19,20,21] algorithms. Kim et al. [8] performed classification of 21 cancer types using gene expressions from The Cancer Genome Atlas project. They compared the neural network, SVM, kNN, and random forest algorithms and reported a higher performance for the neural network. Khan et al. [22] developed a deep learning-based framework for the classification of breast cancers using transfer learning. Prado-Vázquez [19] used a probabilistic graphical model approach for gene expression association and performed the kNN algorithm for breast cancer molecular classification. Some studies report that selecting significant genes (features) and filtering uninformative genes improve the result of the classification and reduce the computational cost, such as the least absolute shrinkage and selection operator (LASSO) regression [14]. Liu et al. [23] combined double RBF kernels with weighted analysis to extract effective features from gene expression data.

More recent studies used deep-learning techniques to automate the feature selection task in cancer classification. Most of these studies used a convolutional neural network as the main backbone in their architecture, such as the deep neural network [24], Neural Architecture Search Network Large (NASNEtLarge) [25], and ensemble convolutional neural network [14]. Gao et al. [26] developed a deep learning-based framework for cancer molecular subtype classification. The deep features learned by their framework capture the biological characteristics associated with each molecular subtype. Tandel et al. [27] provided a review of deep learning-based techniques in brain cancer classification.

There are some challenges with the classification based on gene expression data. First, sequencing generates an enormous number of gene mutations, but not all genes or mutations contribute or relate to cancer prediction, and only a subset of discriminatory genes (driver genes) play a role in the prediction. For example, Endiratta et al. [28] reported that *TP53* is the most commonly mutated gene, and *KMT2C*, *KMT2D*, and *ARID1A* are the most commonly mutated driver genes in the US population. A recent analysis of mutations in human cancers classified 568 genes as driver [29]. Mutations in these genes are likely to cause cellular transformation while mutations in other genes (passenger) may contribute little and even degrade the classification performance. Furthermore, in the discriminatory subset, many of the genes may not have mutations, so they do not carry useful information. These genes will increase complexity of computation for classification or prediction tasks. 

One solution is to use chromosomal rearrangements instead of genes or gene expression data for cancer classification. Chromosomal rearrangements provide fewer but informative data that can be used for classification. Chromosomal rearrangements can be further divided into interCAs (rearrangements between two or more chromosomes) and intraCAs (rearrangements within the same chromosome). IntraCAs are significantly more frequent than interCAs in almost all cancer types [1,6]. In breast cancer, the tally of intraCAs is 68190 versus 18925 for interCAs. In prostate cancer, it is reported as 66411 for intraCAs and 25443 for interCAs, and in pancreatic cancer, the numbers are 43293 for intraCAs and 8330 for interCAs. In this work, we investigated the effectiveness of inter- and intraCAs in cancer prediction versus their frequency using the Catalogue of Somatic Mutations in Cancer (COSMIC). 

There is a high chance of dependency between aberrations in the local neighborhood. Li et al. [1] stated that the structural variants that occur closer together in space and time usually fit into one cluster. This indicates that, generally, but not always, there is a mechanistic link between structural variants within clusters. Thus, gathering information on the other structural variants in the neighborhood needs to be taken into account for analysis of chromosomal rearrangements. This issue was addressed in the proposed work.

There are several databases that have catalogued chromosomal aberrations and gene fusions including the COSMIC [30], Mitelman [31], FARE-CAFÉ [32], TCGA Fusion Gene data portal [33], FusionCancer [34], ChiTaRS [35], dbCRID [36], CoonjoinG [37], HYBRIDdb [38], TICdb [39], and ChimmerDB [40]. COSMIC [30] is a database of somatically acquired mutations found in cancer and is a catalogue of translocations and fusions between gene pairs supplemented by clinical data. The Mitelman Database of Chromosome Aberration and Gene Fusion in Cancer [31] is available for searches related to cases of cytogenetics, gene fusion, structural or recurrent aberrations. This database relates gene fusions and other chromosomal aberrations to tumor characteristics, including karyotype abnormalities associated with tumor types. This database is searchable by a wide variety of fields, such as patient age, authors, gene, tumor histology, tissue type, mutation recurrence, associated clinical features, and cancer types. The Atlas of Genetics and Cytogenetics in Oncology and Hematology database [41] provides information about genes, cytogenetics, and clinical entities in cancer. Latysheva and Babu [42] provided a survey of databases for oncogenetic fusion genes. They also summarized the existing software packages and algorithms for identifying gene fusions from sequencing data.

In this work, we proposed using chromosomal rearrangement endpoints instead of gene expression profiles or mutations for cancer type classification using the COSMIC. To the best of our knowledge, this is the first application of multiclass cancer classification based on chromosomal rearrangement endpoints. Chromosomal rearrangements have complex relationships and cannot be presented well by other traditional data structures. We proposed a graph-based structure framework, GraphChrom, which uses a graph neural network to classify cancer types or subtypes. The graph neural network has a message-passing scheme in which each chromosome is updated according to the information (aberrations) aggregated from the other chromosome’s graph neighborhood. In particular, we used a graph attention network (GAT) which is a measure of the importance of aberrations, defined as how much neighbor CAs influence each chromosome. It means that the output vector from the GAT for each chromosome contains information about the aberrations from other chromosomes, which is a span of n-hop from that chromosome. We also proposed using average graph connectivity, a measure of connectedness, to find the more informative graphs in terms of chromosomal rearrangements for classification. We also hypothesized that the effectiveness of interCAs in cancer classification is higher than that of intraCas, which is inversely proportional to their frequency.

The novelty of this work is several-fold: (A) it develops a graph-based framework to present the complexity of chromosomal aberrations; (B) it predicts cancer types (breast, prostate, and pancreatic) using chromosomal rearrangement endpoints and can be extended to cancer subtype prediction; (C) it extracts highly informative aberrations based on the average graph connectivity; (D) it gathers information about other aberrations in the local neighborhood; and (E) it determines the effectiveness of inter- and intraCAs in cancer classification. 

## 2. Materials and Methods

### 2.1. Catalogue of Somatic Mutations in Cancer (COSMIC) 

The COSMIC [30] is a comprehensive database of somatic mutations in human cancers. This dataset consists of information about genes, mutations, and tumor classification, including the primary tissue and tissue subtype. Initially, we derived chromosomal rearrangement data for 16 cancer types. However, the majority of the cancer types were excluded because either the number of their aberrations in the COSMIC or their generated graphs in our framework were not sufficient for training a graph neural network. This is explained in detail in the following sections. The total number of aberrations for nine cancer types is shown in Figure S1. Thus, the experiments were restricted to the three cancer types only (breast, pancreatic, and prostate) that have the highest number of aberrations and also generated the highest number of graphs in our framework. The data were derived from the COSMIC including “Location From” and “Location To” indicating the first breakpoint “from” and the second breakpoint “to”, sample name, mutation type, and cancer type. Each cancer type includes multiple sample names, and each sample name contains multiple aberrations. Each sample in COSMIC is unique and is considered as a single experiment for this analysis. The mutation types of the samples in the COSMIC are categorized into seven groups, including inverted orientation, noninverted orientation, inversion, deletion, tandem duplication, insertion, and unknown types. Mutations include both intraCA and interCA rearrangements. Interchromosomal rearrangements are all reported as the unknown mutation type in the COSMIC while intrachromosomal rearrangements include all the seven mutation types mentioned above. We processed intra-, inter-, and integrated intra-/interchromosomal rearrangements to assess the effect of each group in prediction. 

### 2.2. Overall Architecture of GraphChrom

The input of the framework is chromosomal rearrangement endpoints (CREs) and the output is a label with probability that defines the cancer type. Overall, the framework consists of several components. The first component is a graph generation module which takes the CREs for each sample name and creates a single graph for that sample. Each sample name can contain one or more aberrations related to the same cancer type. Thus, one graph carries the CRE information belonging to one sample name. The output of this module is a set of graphs equal to the total number of sample names.

**Figure 1:**
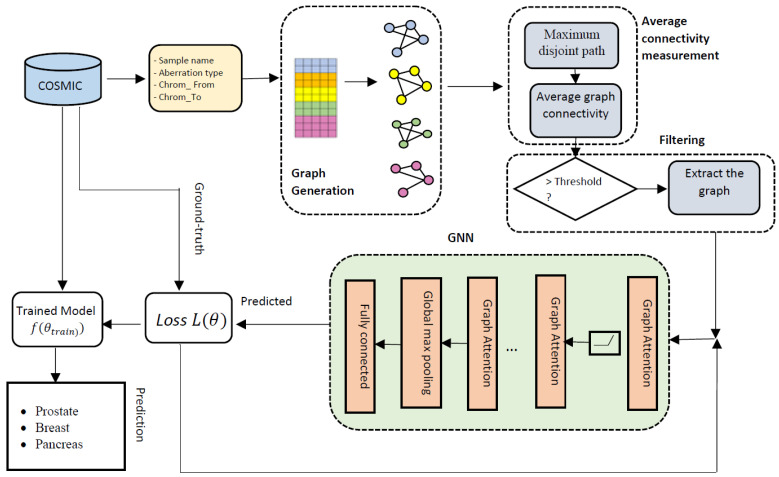
The overall architecture of GraphChrom. The model consists of the following four main components: the graph representation module that generates graphs based on the chromosomal rearrangement endpoints derived from the COSMIC dataset, the average connectivity measurement that computes higher-level information for the graphs, the filtering component that filters out the graphs with the average graph connectivity less than a specified threshold, and the GNN that generates higher-level features of the graphs with the global spatial domain and makes the final prediction.

The graph representations are then fed to the second component to capture the higher level of information including the maximum disjoint paths and the average graph connectivity. The third component is a filtering module to filter out the graphs with the connectivity measure higher than a specified threshold. Finally, there is a classification component, which is a graph neural network that classifies cancer types by taking the filtered graphs as inputs and outputting the predicted cancer type associated with the CREs. The goal of GraphChrom is to predict cancer types using CREs by learning from node embeddings in the graph neural network which is referred to as the graph classification problem. The overall architecture of GraphChrom is shown in Figure 1. The following sections describe each component of GraphChrom in detail. 

#### 2.2.1. CRE Graph Generation

Graphs are a ubiquitous data structure for describing complex systems. In the most general view, a graph is simply a collection of objects (nodes) along with a set of interactions (edges). In this work, graphs were generated based on the CREs derived from the COSMIC dataset. The structure of chromosomal aberrations is formulated as a directed graph *G* (*V*, *E*) where *V* = {1, 2, … *n*} is the set of *n* = 24 chromosomes (nodes) and E denotes the set of rearrangements (edges) between the chromosomes. The graphs are also considered as multigraphs which allows multiple edges between the same pair of nodes, indicating repetition of the same chromosomal aberration for the sample. Each graph is represented as an adjacency list where each element in the list denotes edge $A_{uv}$ that connects node u (source chromosome) to node v (sink chromosome). We referred to the source chromosome as the first breakpoint (“Location From”) and to the sink chromosome as the second breakpoint (“Location To”) in the chromosomal break and used → as the notation to show the direction of the rearrangement. For example, aberration 2 → 3 represents a chromosomal rearrangement from chromosome 2 to chromosome 3. For interCAs, the direction of the edge is from the source chromosome to the sink chromosome. However, for intraCAs, the source and sink chromosomes are the same nodes; thus, graphs contain self-loop edges. An attribute matrix $A\in \;{\mathbb{R}}^{\left|V\right|\times d}$ is created, which represents the edges (chromosomal aberrations) between the nodes. The type of rearrangement (inverted orientation, noninverted orientation, inversion, deletion, tandem duplication, insertion) is unknown for interCAs in the COSMIC, although they are defined for intraCAs.

**Figure 2:**
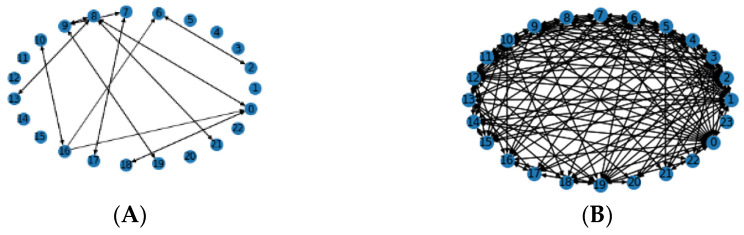
Examples of dense and sparse graphs representing CAs for two distinct samples in (**A**) breast cancer (large number of aberrations (such as 2 → 6, 6 → 2, 16 → 6, 7 → 17, 17 → 7, 8 → 21, 21 → 8, etc.)) and (**B**) prostate cancer (few aberrations (such as 2 → 5, 4 → 10, 17 → 3, 17 → 7, 8 → 21, 21 → 8, etc.)).

The samples contain information including the sample name, sample ID, primary site, mutation type, source chromosome, destination chromosome, etc. All aberrations associated to the same sample name generate one single graph that represents the rearrangement structure for that specific sample. Figure 2 shows two different graphs which are generated based on CREs belong to two different sample names in prostate and breast cancers, respectively. Some samples contain very few aberrations, which develop sparse graphs. It is assumed that these graphs do not carry sufficient information; thus, a strategy is used to extract the graphs that have a higher contribution towards prediction. We proposed using the average graph connectivity using the maximum disjoint paths between the nodes to extract informative graphs, as described in the following subsections. We used only the graphs with the average connectivity higher than a specified threshold in the experiment. The generated graphs in Figure 2 are examples of dense and sparse graphs for breast and prostate cancers.

#### 2.2.2. Maximum Disjoint Path between Two Chromosomes 

According to Menger’s classical theorem [17], in a *k*-connected graph, every pair of nodes are joined by *k* internally disjoint paths. Given digraph G and two nodes, *u* and *v*, disjoint paths are *u*-to-*v* edge paths where no two paths share an edge. We used this theorem to find the maximum disjoint path between every pair of chromosomes in the graph by using a breadth-first search algorithm. The maximum disjoint path between chromosomes is used in the next step for computing the average connectivity of the graphs (described below).

#### 2.2.3. Average Connectivity of CA Graphs

The more connected the graph is (more aberrations in a sample), the more dependencies between the mutations (or rearrangements) there usually (not always) are. We hypothesized that graphs with more links between chromosomes carry more mutation information and play a more important role in prediction than single independent mutations. The measure of how well a graph is connected is connectivity, which is defined as the minimum number of nodes in a set whose deletion results in a disconnected graph. However, since its value is based on a worst-case situation, it does not always reflect the behavior of the whole graph. Thus, we used the average graph connectivity [43], a measure of global graph connectedness, which gives the expected number of nodes that must fail in order to disconnect an arbitrary pair of nonadjacent nodes. In particular, connectivity is a lower bound for the average connectivity. In this work, the average graph connectivity was computed based on the following [43]:

(1) $$\bar{ƙ}\;\left(G\right)=\frac{\sum_{u,v}{ƙ}_{G}\left(u,v\right)}{\left(\begin{array}{c} p\\ 2 \end{array}\right)}$$

where $\bar{ƙ}\;\left(G\right)$ is the average connectivity for graph G, ${ƙ}_{G}\left(u,v\right)$ is the connectivity between chromosome pairs u and v, and p is the order of the graph. In our study, p=24, which represents 24 chromosomes (22 autosomes and two sex chromosomes). We calculated the average connectivity for all the CRE graphs based on the maximum disjoint paths between all pairs of chromosomes.

#### 2.2.4. Filtering

In the filtering module, sparse graphs with the average connectivity below a specified threshold are removed. We used different values using trial and error to find the best threshold that provides the highest performance. In our experiments, it was observed that the prediction accuracy is significantly increased with filtering very dense graphs in which rearrangements have less influence on the structure of the graph. 

#### 2.2.5. Graph Neural Network (GNN)

In GraphChrom framework, the GNN [44,45,46] is the main component that makes the final prediction. GNNs describe a flexible set of architectures for graph-learning tasks and have seen many successful applications over recent years. In this work, the GNN is implemented using the iterative message-passing scheme which allows exchanging vector messages between chromosomes and so update the information of each chromosome as the central node. At each iteration, every node aggregates information from its local neighborhood, and as these iterations progress, each node is embedded with more and more information from further reaches of the graph. In this framework, chromosomes are updated by the other aberrations in the local neighborhood and not only by the immediate neighbors. This is an important factor as structural variants are mechanistically linked to each other [1]. The input of the GNN is the filtered dense graphs from the filtering component, and the output is the predicted cancer type. The graph neural network is trained by computing the loss function between the predicted values and the ground truth data. The proposed GNN includes several layers described as follows.

The graph attention network (GAT) [47] is a version of the graph convolutional network that improves message aggregation by applying attention weights which represent the importance of each chromosome. The measure of importance is defined as how much a neighbor influences the chromosome. Message aggregation in the GAT is defined as follows:

(2) $$m_{\xi }\;\left(u\right)=\underset{v\in \xi }{\sum }\propto_{u,v}h_{v}\;$$

where $\propto_{u,v}$ denotes the attention on neighbor v in the neighborhood of node u when aggregating information at node u and $h_{v}$ is the hidden embedding of node v. The intuition behind using the GAT is to assign an attention weight or importance to each chromosome, which is used to weigh the chromosome’s influence during the aggregation step. Different numbers of GAT layers (radius = n) are used in the GNN architecture. It means that the output vector of the GAT for each chromosome contains information about the aberrations from other chromosomes, which is a span of n-hop from that chromosome. Different values are used for n to explore how strongly the neighborhood CAs influence the CAs on chromosomes. 

The pooling is performed using global max pooling which performs pooling based on the whole graph. It is followed by the last layer of the GAT in the GNN. 

Dense layer—a fully connected layer is created at the last component of the GNN which is followed by the Softmax activation function. 

The objective function is the negative log likelihood loss applied to the log Softmax activation function as follows:

(3) $$loss_{GraphChrom}\left(\theta \right)=-\frac{1}{n}\overset{n}{\underset{i=0}{\sum }}[y_{i\;}\log\left({\hat{y}}_{i}\right)+\left(1-{\hat{y}}_{i}\right)\log(1-{\hat{y}}_{i})]$$

where θ is the set of parameters in the model, n is the total number of graph structures of aberrations, ${\hat{y}}_{i}$ and $y_{i\;}$ are the predicted and ground-truth cancer type values, respectively. 

Adjusting class imbalance: the classes with the imbalanced size of data negatively affect the performance of the classification. In this work, imbalanced numbers of graphs are obtained for different cancer types after applying the filtering module. The list of class sizes for each cancer type is shown in Extended Data Table 1. In order to compensate for the imbalanced data, a weighting strategy is used to oversample the minority classes. The weight of each class is calculated proportionally to the number of samples. Then, the number of samples in each batch are picked up proportionally to the weight of the classes.

### 2.3. Baseline Models

In this section, we described the baseline models used in our experiment, including vanilla networks, SVM, and kNN. The input of these models is the set of all the CREs for the three defined cancer types, in contrast to the GNN in which the inputs are the graphs generated per sample name.

The implemented vanilla network is a three-layer backpropagation neural network with the RELU activation function. The output layer contains three neurons for three-way classification (prostate, pancreatic, and breast cancer). We tested the network with different numbers of hidden layers and neurons and achieved the highest accuracy with three hidden layers and 60 neurons. 

Two experiments were performed using the support vector machine (SVM) based on linear and polynomial functions. Nearly the same performance was achieved for both linear and polynomial SVM kernels. 

The k-nearest neighbors algorithm (kNN) is performed with different numbers of neighbors, from one to hundred, and different similarity measures, including Minkowski, Mahalanobis, and Euclidean distances. 

## 3. Results and Discussion

GraphChrom is implemented in Python 3 using Pytorch 0.40. The training process of the GNN lasts at most 200 epochs using the Adam optimizer with a learning rate of 0.001 and batch size of 10. In this work, n GAT layers were employed, where n varied between 1 and 10, and the 0.2 dropout rate was applied. The total number of aberrations and their corresponding generated graphs in GraphChrom for breast, prostate, and pancreatic cancers are shown in Table 1. These three cancer types have the highest number of samples and aberrations in the COSMIC. This enabled the framework to generate the highest number of graphs for these three cancer types compared to other cancer types.

### 3.1. GraphChrom Predicts Cancer with High Accuracy

**Figure 3:**
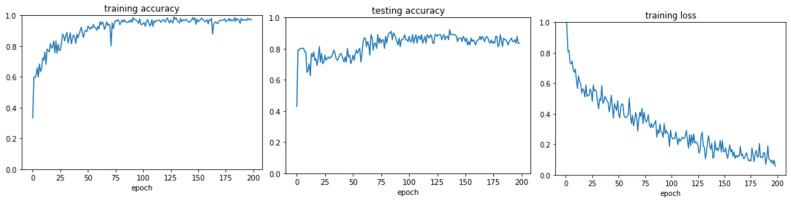
Training accuracy, testing accuracy, and training loss for GraphChrom, 200 epochs, learning rate = 0.001, Adam optimizer, n_hubs = 6.

Overall, 1589 graphs were generated, of which 1137 were filtered out by the filtering module due to sparsity. The size of the training data (inter-, intra-, and integrated CAs) used in the experiment is shown in Figure S2. Different threshold values for the average graph connectivity were tested, and the value with the highest performance (0.3) was chosen. The range of graph connectivity was between 0 and 1. In the experiments, it was observed that most of the aberration graphs had the average connectivity below 0.3. These graphs did not have a sufficient number of endpoints and thus were removed from the experiment. Finally, the model was trained with the remaining 361 and 91 graphs for training and testing, respectively. The imbalanced data were adjusted using a weighting strategy. The training and testing accuracy graphs for GraphChrom are shown in Figure 3. According to the results, a high accuracy classification rate was achieved for all three cancer types. In particular, the classifier obtained 88% overall accuracy for classification. It also achieved 95%, 91%, and 90% accuracy for breast, pancreatic, and prostate cancers, respectively.

### 3.2. Effect of Inter- and Intrachromosomal Aberrations in Prediction

**Figure 4:**
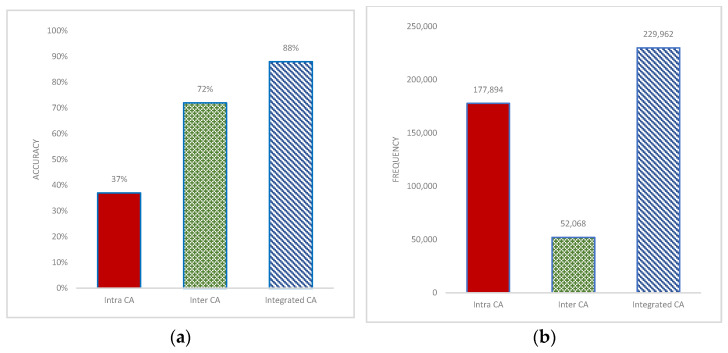
Classification accuracy rate and frequency of aberrations in intrachromosomal, interchromosomal, and integrated aberrations. The frequency is computed based on the data obtained from the COSMIC. Intrachromosomal aberrations are more frequent but have less effect in prediction in contrast to interchromosomal aberrations that are less frequent but significantly affect prediction accuracy. (**a**) classification accuracy for inter CA, intra CA, and integrated CA based on GraphChrom (**b**) Frequency of inter CA, intra CA, and integrated CA.

In this experiment, we investigated how inter-, intra-, and integrated inter-/intraCAs affect the overall prediction. We performed GraphChrom for each of these groups in separate experiments. The results indicate that interCAs were more effective than intraCAs in prediction. The accuracy rate for interCAs was 72% and 37% for intraCAs (Figure 4a). IntraCAs exhibited poor performance and, in particular, did not contribute to prediction on their own. In the integrated experiment, we combined both inter- and intraCAs and applied them to the models. The integrated data provided the highest accuracy of 88% for the overall classification. We also observed that accuracy decreased when removing either inter- or intraCAs. However, the highest decrease could be observed when removing interCAs. This indicates that the predictive effectiveness of interCAs was higher than that of intraCAs, although intraCAs also have a minor role in prediction in the integrated form. 

Surprisingly, the effectiveness of inter- and intraCAs in prediction was inversely proportional to the frequency of aberrations. Although intraCAs occurred at a higher frequency rate than interCAs (Figure 4b), the accuracy of interCAs in prediction was more than double that of intraCAs (Figure 4a). It indicates that the frequency is not the main factor for defining the driver genes/chromosome for cancer prediction. Interchromosomal instability is expected to occur at a lower frequency, perhaps because it is harder to stabilize translocations. In fact, most human genome is “junk” DNA; small intrachromosomal deletions and inversions are tolerated but may not affect cell function. However, certain translocations (e.g., unbalanced) can cause long-range allele loss (e.g., loss of heterozygosity) which could inactivate many genes and cause cell death. In a recent report, we showed that interchromosomal rearrangements are cancer-specific (Mirzaei and Petreaca) while intrachromosomal rearrangements may be “passenger”. Many interchromosomal aberrations occur during DNA replication due to improperly stalled or collapsed replication forks [48]. Recurrent interchromosomal instability was previously shown to correlate with cancer types and in some cases is even used as a diagnostic tool (e.g., the Philadelphia chromosome in certain leukemias and lymphomas) [49]. Thus, it makes sense that the algorithm described in this report forecasts that interchromosomal instability has a higher accuracy of cancer prediction. The frequency of inter- and intra CAs per cancer type is provided in Figure S3. 

### 3.3. GraphChrom Outperforms Baseline Models

**Figure 5:**
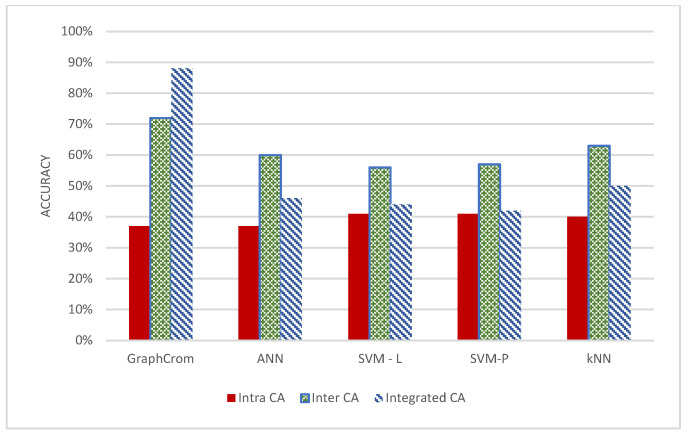
Performance of GraphChrom, vanilla neural network, SVM, and kNN based on inter-, intra-, and integrated chromosomal rearrangement endpoints.

GraphChrom was compared with several baseline models, including the vanilla neural network, SVM-linear, SVM-polynomial, and kNN. In all the models, data were trained based on a training set and evaluated on a testing set. As shown in Figure 5, GraphChrom achieved a higher accuracy rate than the baseline models. In particular, GraphChrom considerably outperformed the baseline models by achieving 88% for integrated CAs compared with less than 50% for the baseline models. 

Surprisingly, in the baseline models, integrated CAs provided a lower accuracy rate compared to interCAs, in contrast to GraphChrom. This is because of the complexity of CAs, and their spatial dependency could be formulated very well with graphs, but not with the baseline classifiers. In fact, GraphChrom has a message-passing strategy that aggregates messages from the neighbor chromosomes to get information about the other CAs. This is an important factor which cannot be addressed in the baseline models. In all the models, intraCAs had a very low contribution to prediction. We also observed that GraphChrom outperformed the baseline models for interCAs with the 73% accuracy rate, compared with the 60%, 56%, 57%, and 63% accuracy rate for the vanilla network, SVM-linear, SVM-polynomial, and kNN, respectively.

### 3.4. Local Chromosomal Aberrations Affect Prediction

In order to investigate how structural variants are mechanistically linked to each other and how they impact prediction, we further performed GraphChrom with different numbers of hubs away from each chromosome, i.e., if we have chain of CAs, how these aberrations are influenced by nonimmediate neighbors. We performed the experiment with different numbers of hubs within GraphChrom. In fact, each chromosome aggregates information not only from direct neighbors but also from neighbors of neighbors in the range of 2–10 hubs away from the original chromosome. For example, the embedding vector of chromosome A consists of its neighbors {B,C,D} when n_hub = 1, and it consists of the second-order neighbors when n_hub = 2 and the third-order neighbors when n_hub = 3, and so on. The more the number of hubs, the more information is incorporated from the chromosome’s neighborhood. Although more information aggregated from the neighborhood can help to increase classification accuracy, at some point, the accuracy begins to decrease. The reason is that one chromosome may appear in different chromosome neighborhoods which causes the same information to be added over and over into the embedding. Additionally, the more there are hubs, the higher the computational complexity. The results indicate that not only the direct neighbors, but also the non-direct neighbors affect prediction. We achieved the maximum performance with six hubs away from each chromosome with 88% accuracy. 

**Figure 6:**
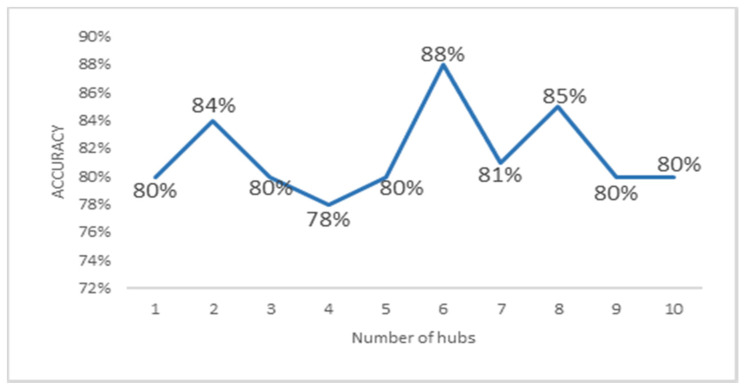
Accuracy of GraphChrom with integrated intra-/interchromosomal aberrations with different numbers of hubs in the GNN.

The accuracy rate obtained for different numbers of hubs in the range of 2–10 is shown in Figure 6. 

### 3.5. Metrics for Classification

The GraphChrom model was evaluated using different performance metrics, including accuracy, precision, recall, and F1-score. Accuracy measures the fraction of correctly classified samples (true samples) to all the samples in the model. Although it is an important metric, it is not sufficient to evaluate a classifier, especially when there are imbalanced data. Precision is the model’s capability of predicting the number of positive samples that are truly positive. In fact, it is the ratio of correctly predicted positive samples (cancer cases) to the total predicted positive samples. Recall or true positive rate (TPR) is the model’s sensitivity and is defined as the ratio of the correctly predicted positive samples (cancer cases) to all cases in the actual class. F1-score is defined as the harmonic mean of the precision and recall of the classifier. Considering the total number of true positive (TP), true negative (TN), false positive (FP), and false negative (FN) results in prediction, performance metrics are defined as follows:

(4) $$Accuracy=\frac{TP+TN}{TP+TN+FP+FN}$$

(5) $$Precision=\frac{TP}{TP+FP}$$

(6) $$Recall=\frac{TP}{TP+FN}$$

(7) $$F1\;score=\frac{2TP}{2TP+FP+FN}$$

**Figure 7:**
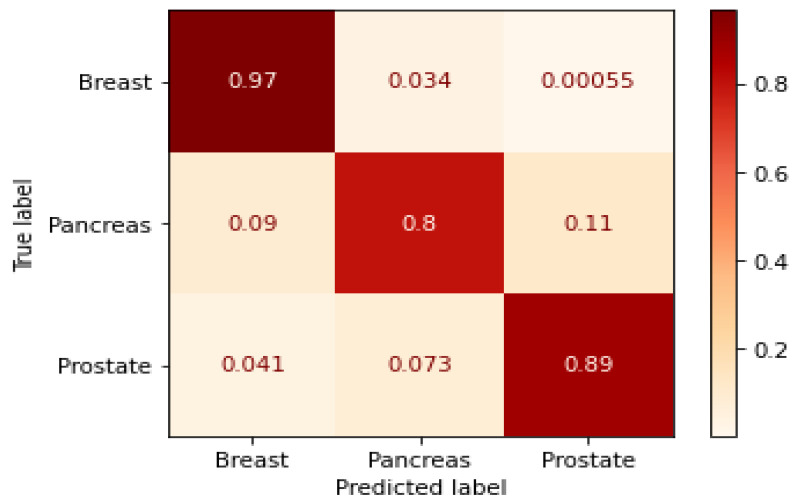
Confusion matrix.

The evaluation metrics and the confusion matrix are shown in Table 2 and Figure 7, respectively. The data fusion matrix is normalized by columns. The actual values of the data fusion matrix are shown in Table S1. GraphChrom achieves the highest accuracy for breast cancer with 95%, followed by pancreatic cancer and prostate cancer with 91% and 90%, respectively. The results show that the model can generalize very well since they become stable as the F1-score and accuracy are high, especially for prostate and breast cancers. 

## 4. Conclusions

In this study, we proposed GraphChrom, a novel graph neural network-based framework for predicting cancer from chromosomal rearrangements endpoints. We performed GraphChrom for predicting breast, prostate, and pancreatic cancers because they have the highest tally of aberrations in the COSMIC. Our model achieved a significantly higher performance in predicting cancer compared with other baseline models. Further, we explored the effect of interCAs, intraCAs, and integrated inter-/intraCAs. 

Moreover, we proposed a novel data extraction method using the average graph connectivity that extracts more informative graphs, leading to effective prediction. We observed that interCAs were more effective in prediction than intraCAs, although the frequency of interCAs was much lower than that of intraCAs. Furthermore, the results show that GraphChrom achieved 88% accuracy in the overall classification, and 95%, 91%, and 90% for breast, pancreatic, and prostate cancers, respectively. The accuracy of GraphChrom outperformed the baseline models. This is the first study that used chromosomal rearrangement endpoints for cancer classification and also investigated the effect of inter- and intrachromosomal rearrangement in prediction. The trained GraphChrom model can be subsequently used for classification of new samples, facilitating clinical implementation of cancer prediction for samples with a large number of aberrations, considering its superior performance. We plan to expand our work by incorporating other cancer types through cross-database experiments.

## Figures and Tables

**Table 1:** The size of the data used in the experiment (number of aberrations and resulting graphs for integrated inter-/intrachromosomal aberrations).

Cancer Type	No. of Aberrations	No. of Graphs
Breast	86,485	598
Pancreatic	51,623	513
Prostate	91,854	478

**Table 2:** Evaluation metrics (%).

Cancer Type	Precision	Recall	F1-Score	Support
**Breast**	0.89	0.96	0.92	30640
**Pancreatic**	0.86	0.8	0.83	26459
**Prostate**	0.91	0.89	0.9	32849
**Accuracy**			0.89	
**Macro average**	0.88	0.88	0.88	89948
**Weighted average**	0.89	0.89	0.89	89948

## Data Availability

The data were obtained from the COSMIC database, which is freely available for noncommercial users. The GraphChrom source code is available at Github (https://github.com/gm932/GraphChrom/blob/main/pytorch_GraphChrom.py, accessed on 3 March 2022).

## Supplementary Materials

The following supporting information can be downloaded at: https://www.mdpi.com/article/10.3390/cancers14133060/s1, Figure S1: Number of aberrations for breast, central nervous system, hematopoietic and lymphoid tissue, liver, lung, ovary, pancreas, prostate, and skin reported on COSMIC, Figure S2: Size of the training data for inter, intra, and integrated chromosomal aberrations for breast, prostate, and pancreas cancers (number of graphs generated by GraphChrom), Figure S3: Frequency of chromosomal rearrangement per cancer type, Figure S4: GAT architecture based on attention mechanism that indicates the importance of node j’s feature to node i, $e_{ij}=a(W\vec{h_{i}},\;W\vec{h_{ij})}$ where $e_{ij}$ is the attention coefficients, ***W*** is a weight matrix, and $h_{i}$, $h_{j}$ are node features, Table S1: Confusion matrix with actual values.
