# Supplementary material for: GraphChrom: A Novel Graph-Based Framework for Cancer Classification Using Chromosomal Rearrangement Endpoints
Source: Cancers (Basel). 2022 Jun 22;14(13):3060. doi: 10.3390/cancers14133060 (PMC9265123; doi:10.3390/cancers14133060)
Supplement: Supplementary file 1 [file cancers-14-03060-s001.zip › cancers-1710608-supplementary.pdf]

Figure S1. Number of aberrations for breast, central nervous system, hematopoietic and lymphoid tissue, liver, lung, ovary, pancreas, prostate, and skin reported on COSMIC

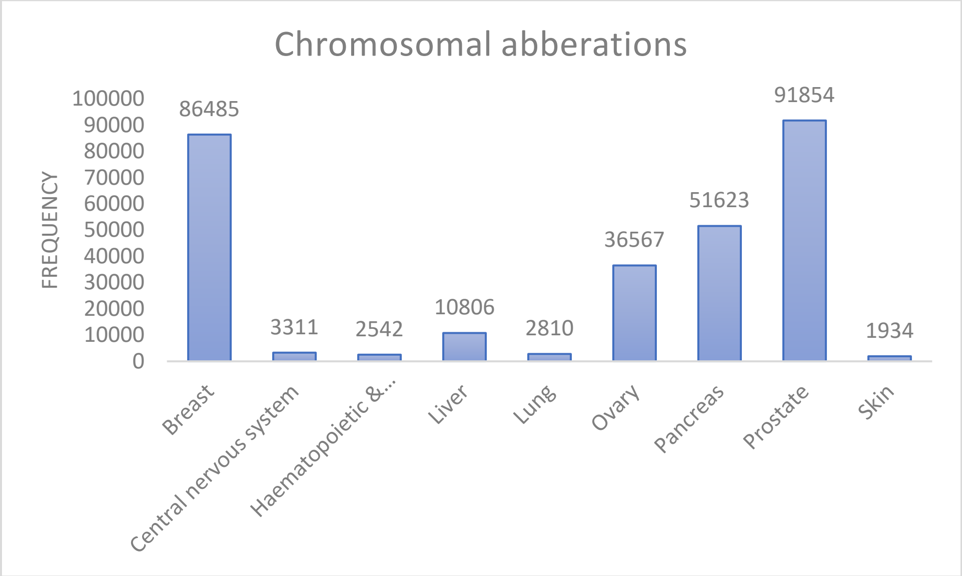

Figure S2. Size of the training data for inter, intra, and integrated chromosomal aberrations for breast, prostate, and pancreas cancers (number of graphs generated by GraphChrom)

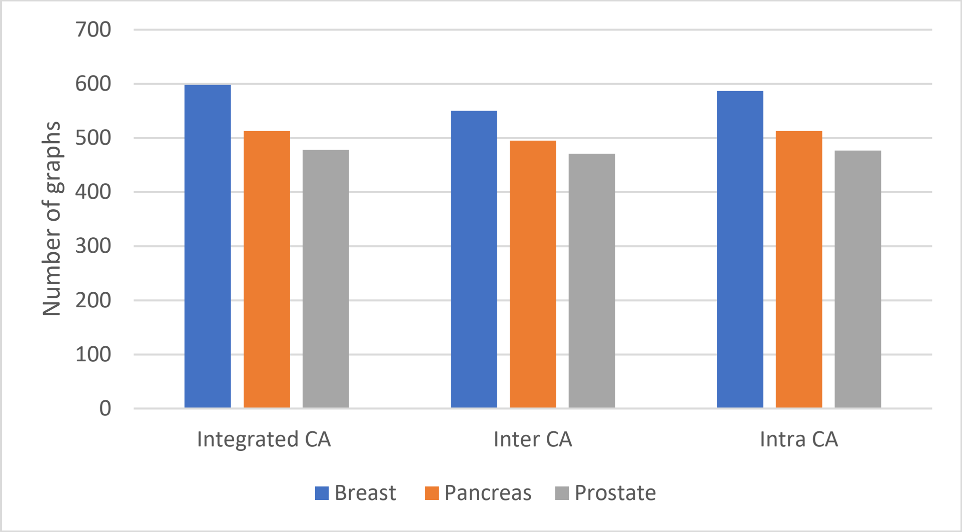

Figure S3. Frequency of chromosomal rearrangement per cancer type

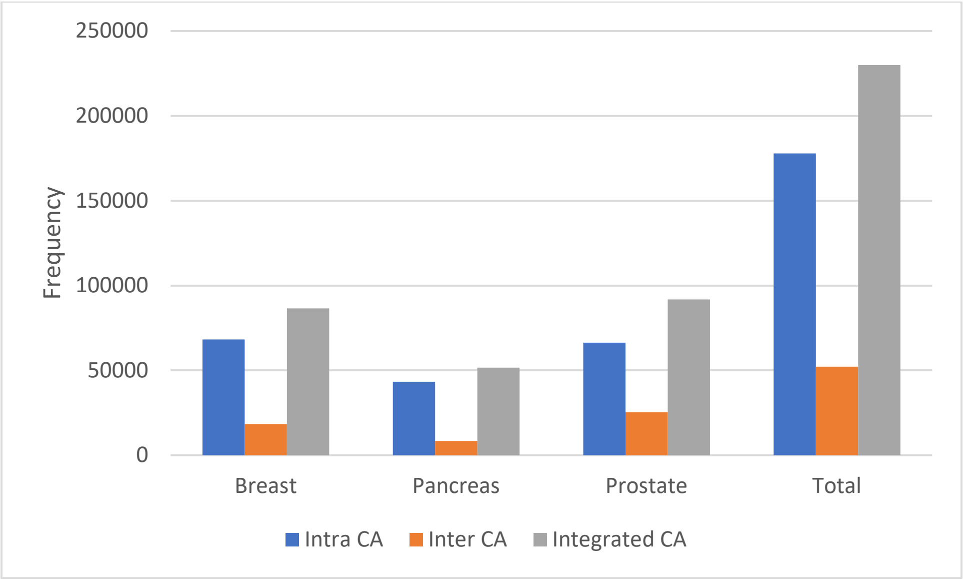

Figure S4. GAT architecture based on attention mechanism that indicates the importance of node j's feature to node i , $e_{ij} = a(\overrightarrow{\boldsymbol{W}h_i}, \overrightarrow{\boldsymbol{W}h_j})$ where e_{ij} is the attention coefficients, $\boldsymbol{W}$ is a weight matrix, and h_i , h_j are node features. The attention mechanism α is a single-layer feedforward neural network parameterized by a weight vector and applying the RELU nonlinearity.

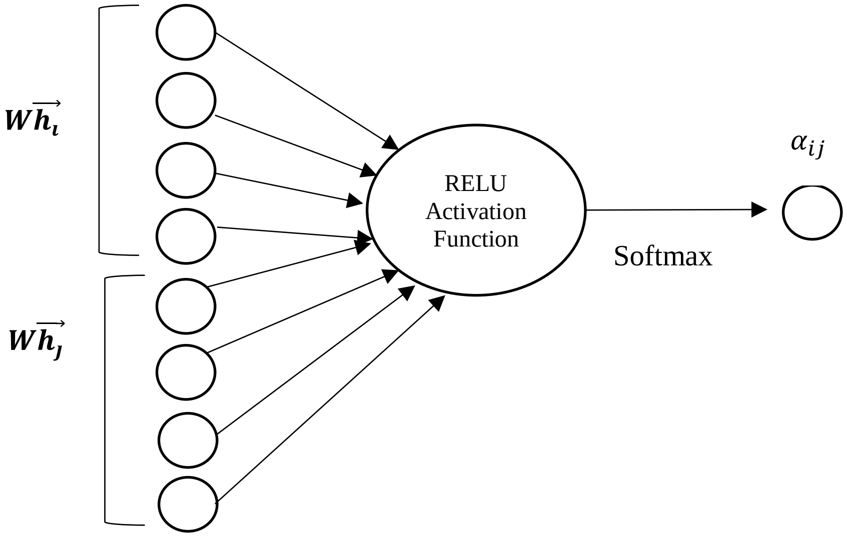

Table S1. Confusion matrix with actual values

	Breast	Pancreas	Prostate
Breast	29579	1044	17
Pancreas	2377	21120	2962
Prostate	1350	2388	29111
